# β1 integrins mediate the BMP2 dependent transcriptional control of osteoblast differentiation and osteogenesis

**DOI:** 10.1371/journal.pone.0196021

**Published:** 2018-04-20

**Authors:** Molly Brunner, Noémie Mandier, Thierry Gautier, Genevieve Chevalier, Anne-Sophie Ribba, Philippe Guardiola, Marc R. Block, Daniel Bouvard

**Affiliations:** 1 Centre de Recherche INSERM 1209, CNRS 5309, Institute for Advanced Bioscience; Université Grenoble Alpes, Grenoble, France; 2 Centre Hospitalier Universitaire and University of Angers, SNP Plateform, Institute for Biological Health, Transcriptome and Epigenomic, Angers, France; Universite de Nantes, FRANCE

## Abstract

Osteoblast differentiation is a highly regulated process that requires coordinated information from both soluble factors and the extracellular matrix. Among these extracellular stimuli, chemical and physical properties of the matrix are sensed through cell surface receptors such as integrins and transmitted into the nucleus to drive specific gene expression. Here, we showed that the conditional deletion of β1 integrins in the osteo-precursor population severely impacts bone formation and homeostasis both *in vivo* and *in vitro*. Mutant mice displayed a severe bone deficit characterized by bone fragility and reduced bone mass. We showed that β1 integrins are required for proper BMP2 dependent signaling at the pre-osteoblastic stage, by positively modulating Smad1/5-dependent transcriptional activity at the nuclear level. The lack of β1 integrins results in a transcription modulation that relies on a cooperative defect with other transcription factors rather than a plain blunted BMP2 response. Our results point to a nuclear modulation of Smad1/5 transcriptional activity by β1 integrins, allowing a tight control of osteoblast differentiation.

## Introduction

Proliferation, differentiation and survival of adherent cells are all tightly dependent on cell interaction with the extracellular matrix (ECM) or neighboring cells. Therefore, the nature and the physical proprieties of the extracellular matrix are important players during mammalian development and tissue integrity. During bone formation and homeostasis, osteoblasts are responsible for the deposition of novel bone matrix at sites of osteoclast dependent bone resorption [1]. New bone matrix synthesis is a direct response to changes that occur within the surrounding environment. While not totally understood, an emerging hypothesis is that the remodeled bone matrix impacts on both the physical quality (stiffness) and composition of the bone with the release of ECM fragments and growth factors [2]. Matrix stiffness and resulting cellular tension have emerged to be critical for mesenchymal stem cell fate and proliferation [3]. Recently, it has been shown that mesenchymal stem cells are able to integrate the extracellular environment stiffness to control YAP/TAZ signaling and thereby cell proliferation and differentiation [4]. Additionally, soluble growth factors such as BMPs, WNTs or PTHrP also play a pivotal role to control proper osteoblast proliferation and differentiation [5]. Among those soluble factors, BMP2 is of prime interest in this latter process. Indeed, BMP2 deletion during development leads to a severe defect in post-natal bone formation [6]. Likewise, BMP receptor 1A (BMPR1A) or SMAD-4 (a BMP effector) deletions in mature osteoblasts, as well as the overexpression of the BMP-inhibitor Noggin, led all to a significant reduction in osteoblast activity [7, 8].

These mechanical and matrix associated cues are likely very important for controlling osteoblast/osteoclast coupling as mentioned above as well as developmental and pathological processes. During bone development, pre-osteoblasts are located both within the connective tissue (periosteous) and at mineralized surfaces, while differentiated osteoblasts are exclusively found in close contact to mineralized bone matrix [9]. These findings favor the view that matrix rigidity may provide a signal necessary for pre-osteoblast to osteoblast transition.

Integrins as the main receptors for cell adhesion to the ECM are likely important mechano-transducers in these processes. Although lacking any enzymatic activity, they are crucial for bidirectional signaling. In one hand, they are involved in matrix organization and deposition [9–11] thereby creating a signaling niche that facilitate outside-in signaling from cognate receptors such as growth factors receptors. On the other hand, integrin affinity and clustering are regulated from an inside-out signaling pathway. Fibronectin (FN) through its interaction with α5β1 integrins, as well as type I collagen, was shown to be necessary for osteoblast differentiation and survival [12–14], while the deletion of β1 integrin specific inhibitor such as ICAP-1 was reported to affect bone formation in mice [15]. Mechanistically, ICAP-1, by preventing an excessive talin and kindlin-2 recruitment on β1 integrin cytoplasmic tails, favors β1 integrin cycling between low and high affinity that is required for fibrillogenesis and collagen deposition [11, 16]. Finally, it was shown that α5β1 activation by an agonist peptide also promotes osteoblast differentiation through the activation of a FAK and MAPK/ERK-dependent pathway [17, 18]. Unexpectedly, the conditional deletion of β1 integrins in mature osteoblasts using the type I collagen (Col 2.3) promoter driving Cre recombinase expression led to a relatively slight phenotype in mice [19]. Since, the expression of a dominant negative (DN) form of β1 integrins under the control of the osteocalcin (OCN) promoter that targets mature osteoblasts demonstrated a limited role for these integrins in this cell type [20], the absence of a severe bone phenotype in the former mouse model may be explained by the relatively late deletion of β1 integrins. This hypothesis was further confirmed by the analysis of conditional deletions of β1 integrins at different developmental stage during osteogenesis [21].

In this study, we analyzed the role of β1 integrins at an early stage of osteoblast differentiation using the Osterix (Osx) promoter to drive Cre recombinase expression (β1^Ost-ko^ mice). Interestingly, in pre-osteoblasts, the β1 integrin deletion led to a severe bone mass reduction in young mice as previously reported [21]. However, it was not reported how integrins affect osteoblast differentiation at the molecular level. We showed that, mechanistically, this phenotype was associated *in vivo* with a clear decrease in type I collagen deposition and Smad1/5 phosphorylation. Using *in vitro* osteoblast cultures, we demonstrated that β1 integrins deletion neither affects Smad1/5 phosphorylation *ex vivo* nor their nuclear localization, but rather acts downstream into the BMP signaling pathway, likely by regulating the establishment of a proper network of transcription factors involved in the transition between pre-osteoblast to osteoblast.

## Results

### Efficiency of β1 integrin deletion by Osx-Cre deletor mice

**Fig 1:**
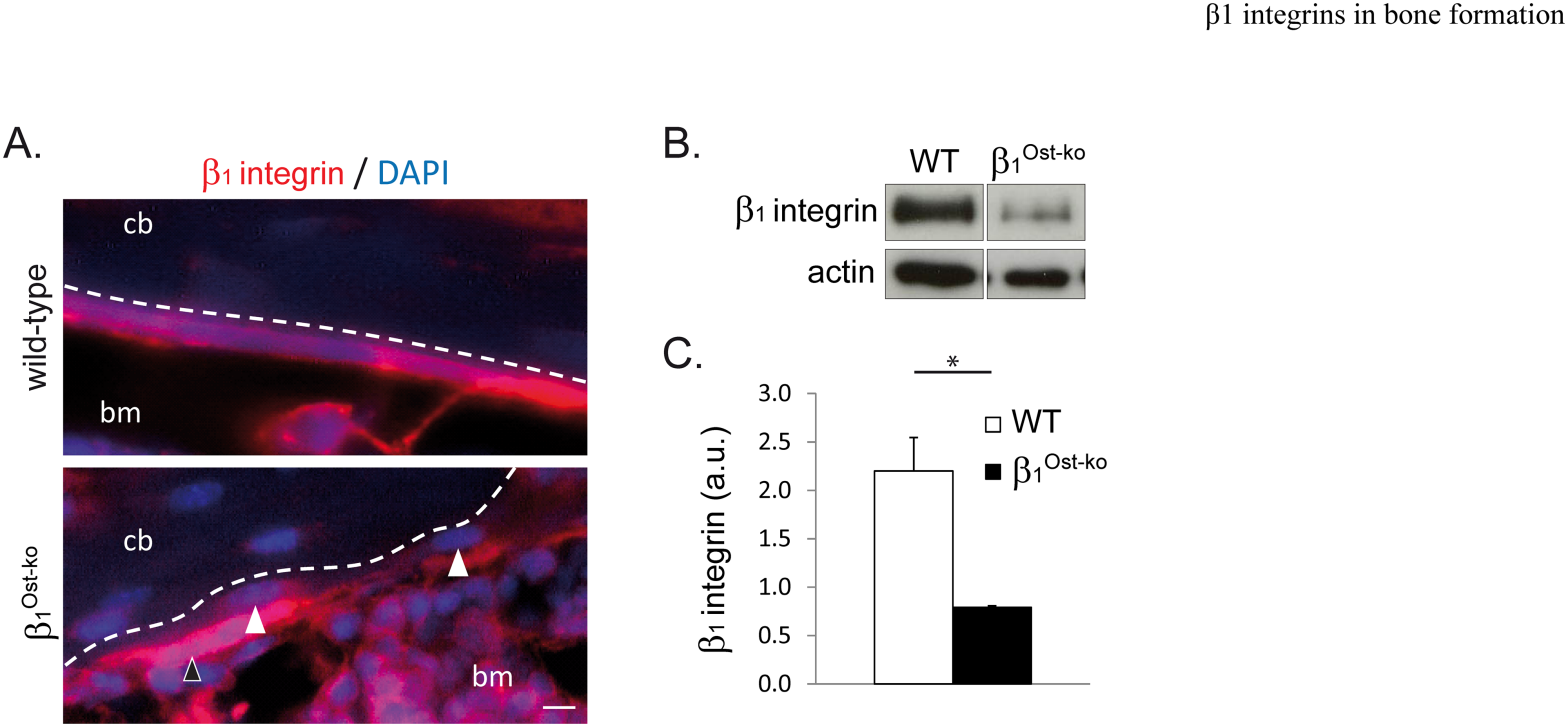
β1 integrin deletion in β1^Ost-ko^ mice. **A.** Immunofluorescence analysis of β1 integrin expression on wild-type and β1^Ost-ko^ P4 mice sections (cb: cortical bone; bm: bone marrow; dash line represents cortical the bone surface; arrows show osteoblasts deleted of β1 integrins; scale bar represents 10μm). Note that β1 integrin deletion is not total in β1^Ost-ko^ mice. **B and C.** Western-blot analysis and quantification of β1 integrin expression in long bones lysates from wt and β1^Ost-ko^ P30 mice. β1 integrin expression was normalized with actin (*: p<0.05).

Since, β1 integrins have a limited role in mature osteoblasts, we hypothesize that deletion using the Col1-Cre deletor mice might occur at a stage too late to impair the osteoblast differentiation and reveal any important function of β1 integrins in this process. To specifically address this question, we crossed the Osterix-Cre (Osx-Cre) deletor mice with mice bearing β1 integrins floxed alleles [22]. To characterize the β1 integrin deletion using the Osx-Cre deletor mice, we performed immunofluorescence staining on femur from 4-days old WT and β1^Ost-ko^ mice. As expected, a clear reduction in the number of β1 integrin-positive osteoblasts at the surface of cortical bone (Fig 1A, white arrow heads) was observed. This was also accompanied by an increase in β1 integrin-negative osteocytes embedded into cortical bone. β1 integrin deletion was then quantified by Western-blotting of long bones lysates from wild-type and β1^Ost-ko^ mice (Fig 1B). As expected, we observed a clear reduction in β1 integrin expression in β1^Ost-ko^ mice compared to wild-type. Western-blotting quantification and normalization with actin allowed us to estimate a 2.78-fold reduction in β1 integrin expression in β1^Ost-ko^ mice compared to WT (Fig 1B and 1C). This incomplete deletion of β1 integrins in β1^Ost-ko^ mice can be explained by the presence of progenitors that still do not express the Osterix transcription factor or by the partial recombinase deletion of the β1 integrin gene. Nevertheless, the deletion was sufficient to induce a drastic phenotype in β1^Ost-ko^ mice, highlighting the important role of this integrin class in bone formation and homeostasis.

### β1 integrins are important regulators of bone formation at the pre-osteoblastic stage

**Fig 2:**
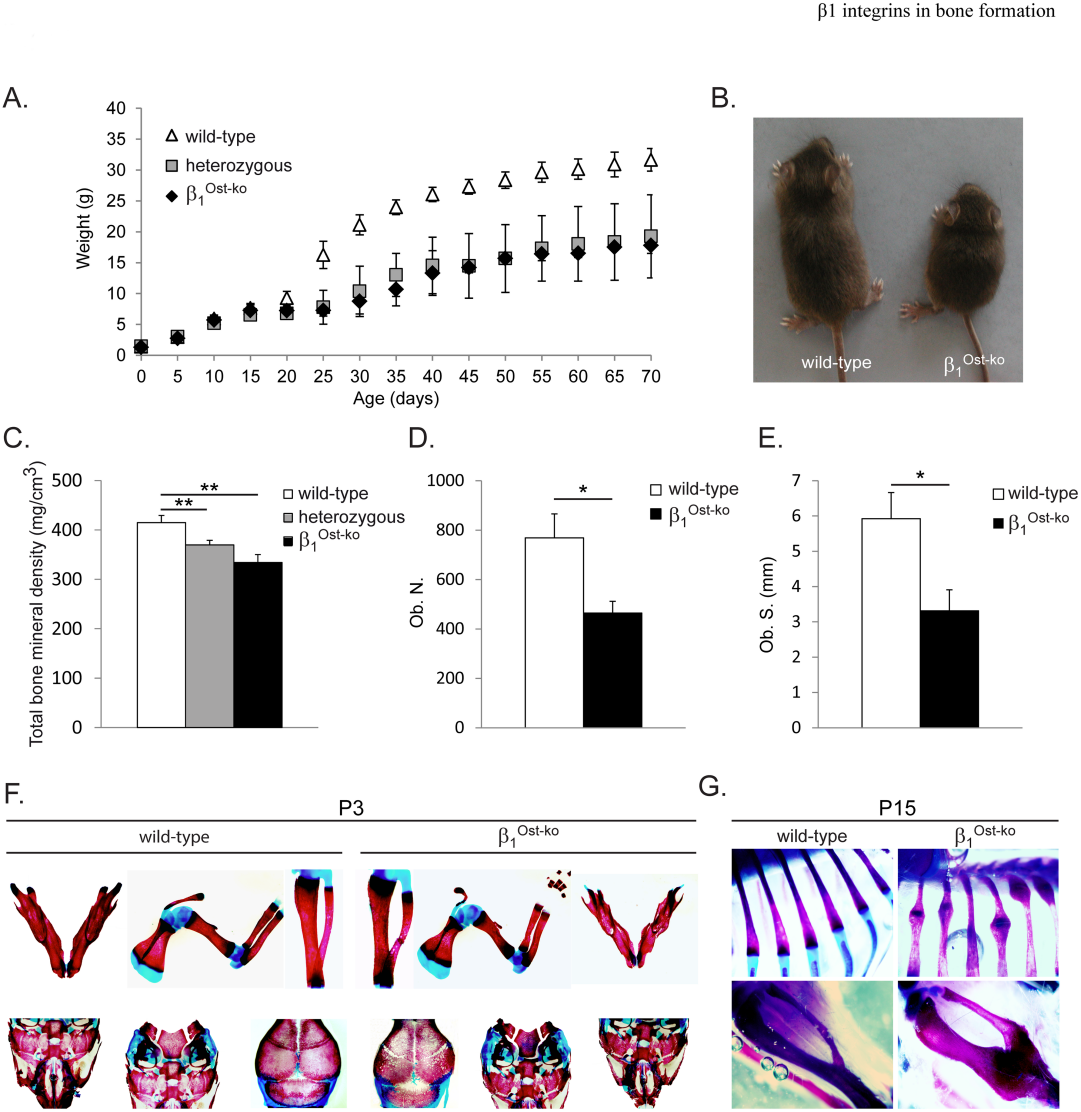
β1 integrin deletion at pre-osteoblastic stage leads to severe bone phenotype. **A.** Growth curve of wild-type, heterozygous and β1 integrin deleted (β1^Ost-ko^) mice. **B.** Picture of wild-type and β1^Ost-ko^ mice showing the growth defect due to β1 integrin deletion at pre-osteoblastic stage. **C.** Total bone mineral density (BMD) of wild-type, heterozygous and mutant mice measured using micro Computed Tomography (**: p<0.005). **D and E.** Histomorphometric analysis of osteoblast number (Ob. N.) **D.** and surface (Ob. S.) **E.** at P30 depending on mice genotype (*: p<0.05). **F and G.** Alizarin Red and Alcian Blue staining of skeleton from 3-days or 15-days old wild-type and β1^Ost-ko^ mice. Note the increased porosity of skull, jaw and long bones, as well as bending and hypertrophic callus resulting from former fractures in β1^Ost-ko^ mice.

Mutant mice deleted for β1 integrins (β1^Ost-ko^ mice) as well as heterozygous mice (Ost-Cre+; β1^+/f^) were significantly smaller upon aging than wild-type mice (Fig 2A and 2B). Interestingly, this growth defect was more visible after weaning (post-natal day 21, P21). When looking specifically at the bone tissue, mutant and heterozygous mice displayed a significant reduction in bone mineral density measured by micro Computed Tomography (Fig 2C). Histomorphometric analyses of β1^Ost-ko^ mice showed a reduced number of osteoblasts and a reduced bone surface covered by osteoblasts compared to their wild-type littermates (Fig 2D and 2E). This was accompanied by a strong mineralization defect, increased porosity and fragility of long bones as well as flat bones such as calvaria and scapula at P3 (Fig 2F). This occasionally leads to fractures, bending, and hypertrophic callus resulting from prior fractures in β1^Ost-ko^ mice long bones such a ribs and tibia (Fig 2G, upper and lower panel respectively). All together these data highlighted the importance of β1 integrins at early stages of osteoblast differentiation for proper bone formation as previously reported (21).

### β1 integrin deletion leads to reduced Type I Collagen deposition and mineralization

**Fig 3:**
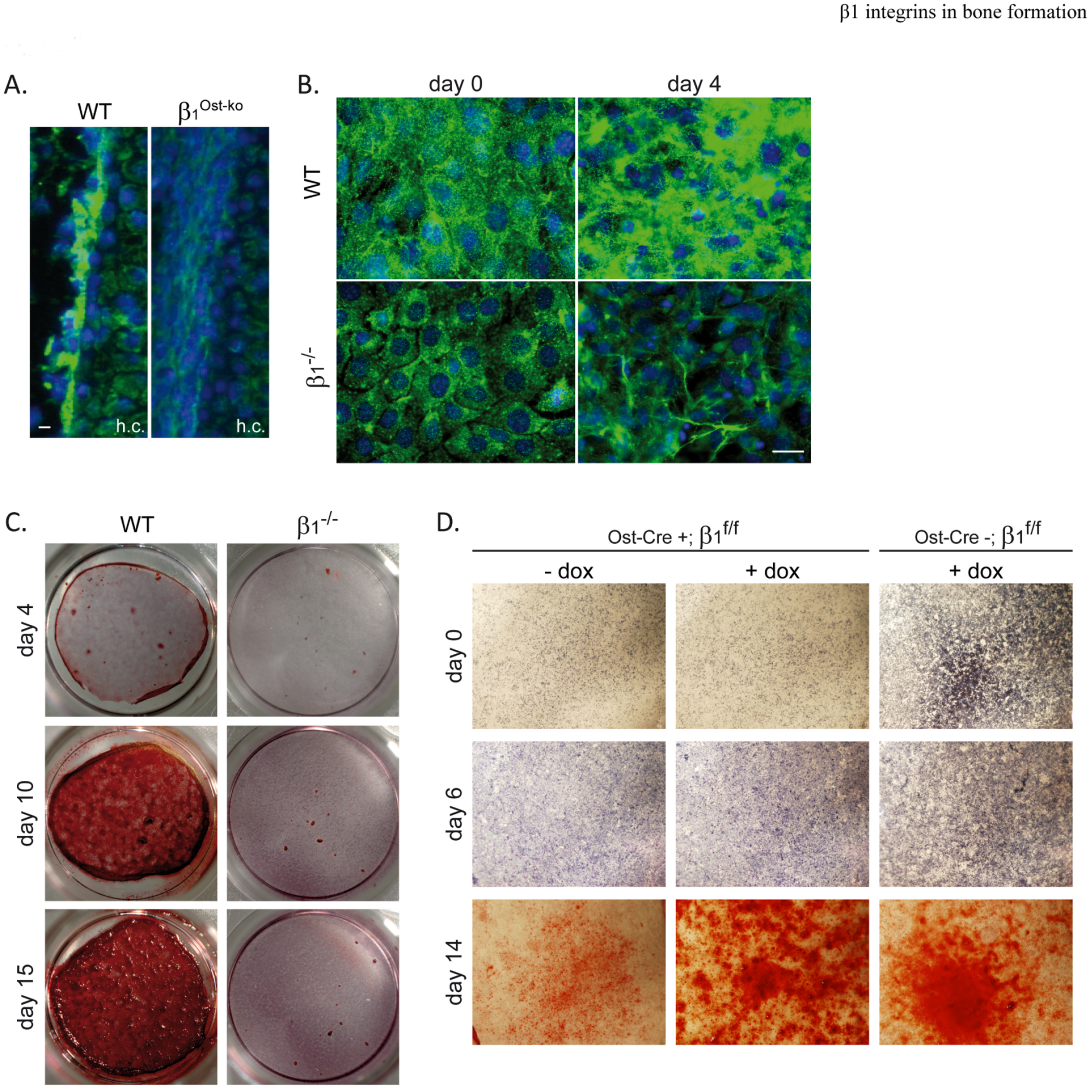
β1 integrins are necessary for Type I Collagen deposition and mineralization. **A.** Immunofluorescence analysis of Type I Collagen deposition on wild-type and β1^Ost-ko^ E14.5 embryos femur sections (h.c.: hypertrophic cartilage; scale bar represents 10μm). Note that Type I Collagen deposition onto β1^Ost-ko^ developing bones surface was almost absent. **B.** Immunofluorescence staining of Type I Collagen deposition by wild-type and β1 deleted (β1^-/-^) immortalized osteoblasts induced to differentiate for 4 days (day 4) or not (day 0)(Scale bar represent 10μm). **C. and D.** Mineralization capability, visualized by Alizarin red staining, of WT vs β1^-/-^, and Ost-Cre expressing β1^f/f^ vs control (Ost-Cre negative) immortalized osteoblasts induced to differentiate for 4, 10 or 15 days (C.) or 0, 6 and 14 days (D.). Doxycycline was added to the medium (+dox) to block Cre expression. Note the lack of mineralization of β1^-/-^ and Ost-Cre+;β1^f/f^ osteoblasts cultures and the mineralization rescued in presence of doxycycline.

We previously reported the role of β1 integrin activation/de-activation cycle in fibronectin and collagen matrix deposition, a prerequisite for mineralization (11). Hence, we analyzed matrix deposition capability of osteoblasts deleted for β1 integrins. Immunofluorescence staining for Type I Collagen on femur sections of E14.5 WT or β1^Ost-ko^ embryos were carried out and revealed that β1 integrin deletion strongly impaired Type I collagen deposition at the periosteum surface of forming bones (Fig 3A). The collagen deposition defect was also confirmed *ex vivo* using deleted (β1^-/-^) or control immortalized osteoblasts induced to differentiate in a conditioned medium. At confluence, wild-type osteoblasts showed an important Type I Collagen deposition that increased after 4 days of differentiation. In contrast, β1^-/-^ osteoblasts exhibited a reduced collagen deposition at confluence that did not increase significantly during differentiation (Fig 3B). We previously demonstrated that mineralization requires a proper matrix deposition by osteoblasts. Accordingly, differentiation in a conditioned medium for 4, 10 or 15 days revealed a complete absence of mineralization of β1^-/-^ osteoblasts visualized by Alizarin red staining. These results were validated with 3 independent clones to rule out any clonal effect on differentiation capability and demonstrated the importance of β1 integrins for proper Type I Collagen matrix deposition and subsequent mineralization by osteoblasts. Moreover, mineralization capability of osteoblasts isolated from wild-type (Ost-Cre-; β1^f/f^) or mutant (Ost-Cre+; β1^f/f^) mice confirmed that β1 integrins deletion during osteoblast differentiation impairs osteoblast mineralization and that blocking Cre expression with doxycycline rescued the mineralization phenotype in Ost-Cre+; β1^f/f^ (Fig 3D).

### β1 integrins are essential for Smad1/5 phosphorylation in vivo

**Fig 4:**
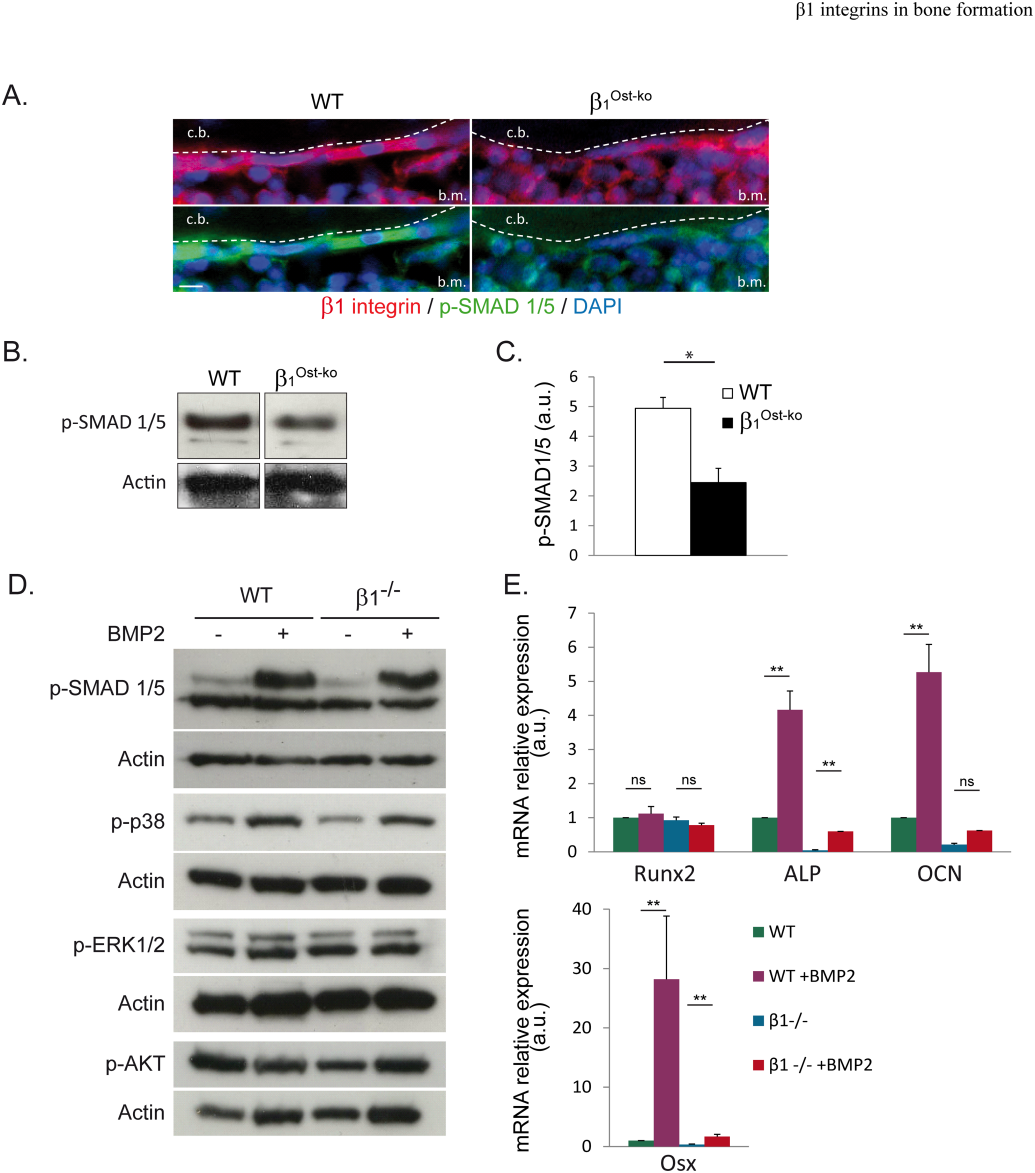
β1 integrins are essential for Smad1/5 phosphorylation *in vivo*, but controls the expression of BMP2 targeted genes regardless of Smad1/5 phosphorylation *ex vivo*. **A.** Immunofluorescence analysis of β1 integrins as well as Smad1/5 phosphorylation (P-Smad1/5) on wild-type and β1^Ost-ko^ P4 mice sections (c.b.: cortical bone; b.m.: bone marrow; scale bar represents 10μm). Note the almost absence of Smad1/5 phosphorylation in the lack of β1 integrins. **B and C.** Western-blot analysis and quantification of Smad1/5 phosphorylation in long bones lysates from wt and β1^Ost-ko^ P30 mice. Smad1/5 phosphorylation was normalized with actin (*: p<0.05). **D.** Western-blot analysis of Smad1/5, p38, ERK1/2 and AKT phosphorylation in immortalized WT and β1^-/-^ osteoblasts cultures treated or not with BMP2. **E.** Quantitative PCR analyses of BMP2-independent Runx2, or BMP2-dependent Alkaline Phosphatase (ALP), Osteocalcin (OCN) and Osterix (Osx) mRNA expression levels in WT or β1^-/-^ osteoblasts cultures treated or not with BMP2 (*: p<0.005). Note the absence of induction by BMP2 of target genes in β1^-/-^ osteoblasts.

Next we wondered whether the collagen deposition defect alone accounted for the bone phenotype. A major osteogenic factor regulating Alkaline Phosphatase (ALP), Osterix (Osx) expression, and involved in bone development is the Bone Morphogenetic Protein 2 (BMP2) [6]. Some *ex vivo* studies demonstrated a link between BMP2 and integrin signaling [9, 13, 23]. Moreover, the expression of a dominant negative BMP2 receptor in maturing osteoblasts partly phenocopied β1 integrin deletion in pre-osteoblasts [24]. However, it is still not clear whether the crosstalk between BMP2 and integrins is relevant *in vivo*, and which mechanisms are implicated. Thus, we asked whether the drastic bone phenotype induced upon β1 integrin deletion at the pre-osteoblastic stage *in vivo* could be linked to any BMP2 signaling defect. To address this important question, we compared Smad1/5 phosphorylation *in vivo* in wild-type and β1^Ost-ko^ mice. As revealed by immunofluorescence, wild-type osteoblasts at the cortical bone surface were strongly positive for p-Smad1/5 staining, while on β1^Ost-ko^ mice sections, this staining was almost absent (Fig 4A). These results were confirmed by Western-blotting of bone lysates showing a 2-fold reduction in Smad1/5 phosphorylation in β1^Ost-ko^ compared to WT mice (Fig 4B and 4C). Therefore, these data demonstrated the positive control of β1 integrins on Smad1/5-dependent BMP2 signaling *in vivo*.

### β1 integrins are necessary for the expression of BMP2 targeted genes independently on Smad1/5 phosphorylation

**Fig 5:**
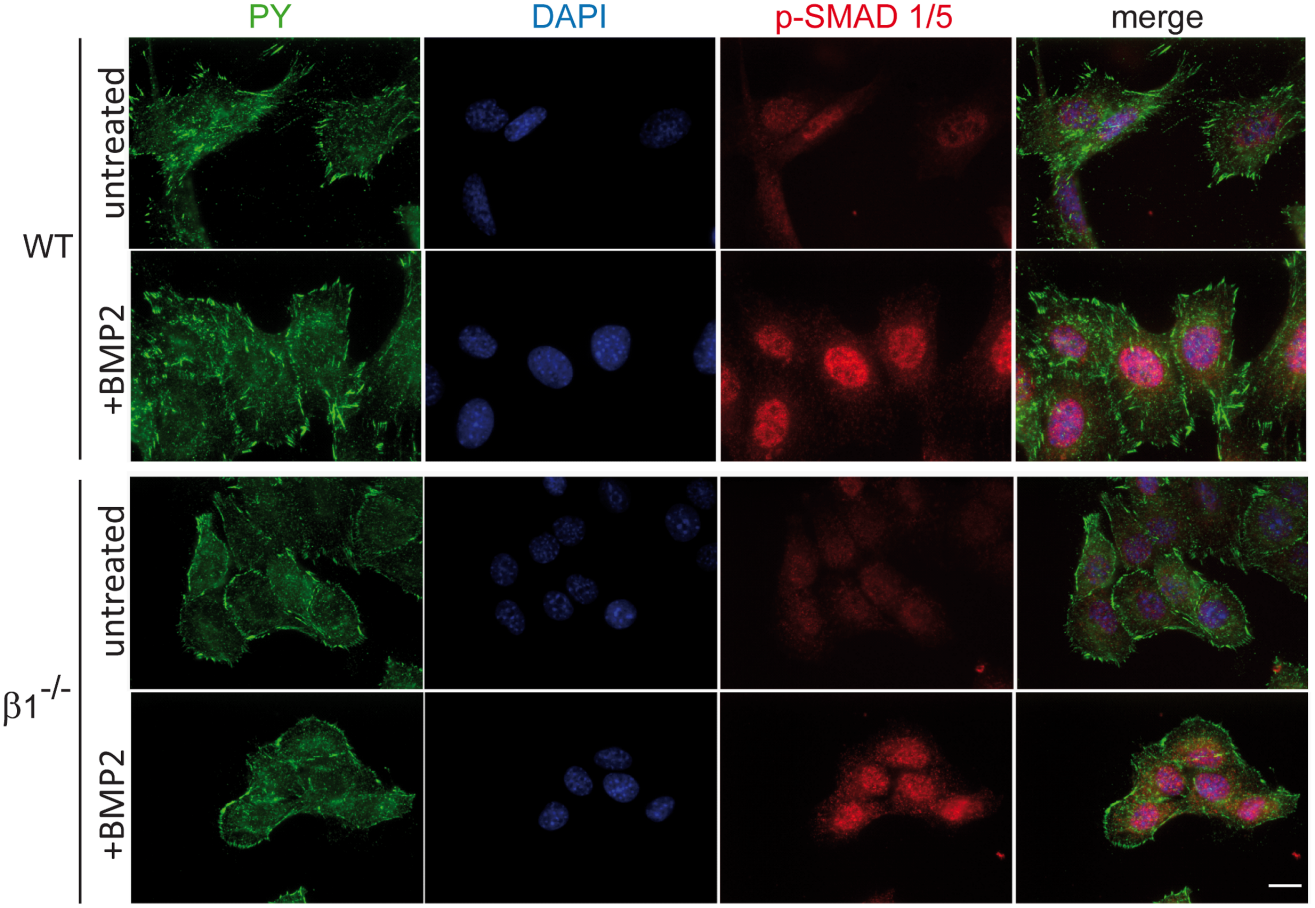
Efficient Smad1/5 nuclear translocation in absence of β1 integrins. Smad1/5 nuclear translocation was analyzed using immunofluorescence under basal conditions (untreated) or after BMP2 treatment (+BMP2; PY: phospho-tyrosine; scale bar represents 10μm). Note the clear P-Smad1/5 nuclear translocation after BMP2 treatment in WT, as well as β1 deleted (β1^-/-^) osteoblasts.

As mentioned above, previous *ex vivo* studies demonstrated a link between integrins and BMP2 receptor activation [23]. However, it is not clear in bone at which level β1 integrins act to favor BMP2-dependent signaling. In order to further characterize the input of β1 integrins in the BMP2/Smads pathway, we studied BMP2 downstream signaling in immortalized osteoblasts cultures deleted or not for β1 integrins. Unexpectedly, when analyzing the phosphorylation of key proteins of main signaling pathways, such as p38, Smad1/5, ERK1/2 or AKT, in response to BMP2, no major differences were observed between WT and β1^-/-^ cells (Fig 4D). Interestingly, the previously observed Smad1/5 phosphorylation defect upon β1^-/-^ osteoblasts removal *in vivo* was no longer reproduced *ex vivo* after stimulation with BMP2. Indeed, treatment with BMP2 led to a significant Smad1/5 phosphorylation in both WT and β1 deleted cells (Fig 4D, upper panel). Likewise, p38 phosphorylation in response to BMP2 was not affected by β1 integrin loss, while ERK1/2 phosphorylation remained unchanged regardless of the expression or β1 integrins. Conversely, we noted a slight reduction in AKT phosphorylation after BMP2 treatment that was not reproduced in β1^-/-^ cells. To further investigate β1^-/-^ osteoblasts response to BMP2 treatment *ex vivo*, we analyzed the final events of BMP2-dependent pathway activation, i.e. the expression of BMP2 target genes. Unexpectedly, quantitative PCR analyses revealed that the expression of BMP2 target genes (ALP, OCN and Osx) was clearly reduced in response to BMP2 treatment in β1 deleted compared to WT osteoblasts (Fig 4E). One explanation for the apparent discrepancy between efficient Smad1/5 phosphorylation and the absence of target genes expression in the absence of β1 integrins may be the lack of efficient nuclear translocation of Smads in β1^-/-^ osteoblasts. Indeed, it was previously reported that integrins can regulate the nuclear translocation of signaling proteins such as ERK or α-NAC [9, 25, 26]. To test this hypothesis, we performed immunofluorescence analyses of p-Smad1/5 subcellular localization under basal conditions or after BMP2 treatment. Importantly, WT as well as β1^-/-^ osteoblasts exhibited a clear nuclear translocation of Smad1/5 in response to BMP2 (Fig 5).

These results led us to conclude that β1 integrins are important regulators of BMP2/Smads signaling *ex vivo*, but not at the level of BMP receptor activation or Smad phosphorylation but rather downstream in the signaling pathway.

### β1 integrins regulate a subset of genes involved in bone mineralization

**Fig 6:**
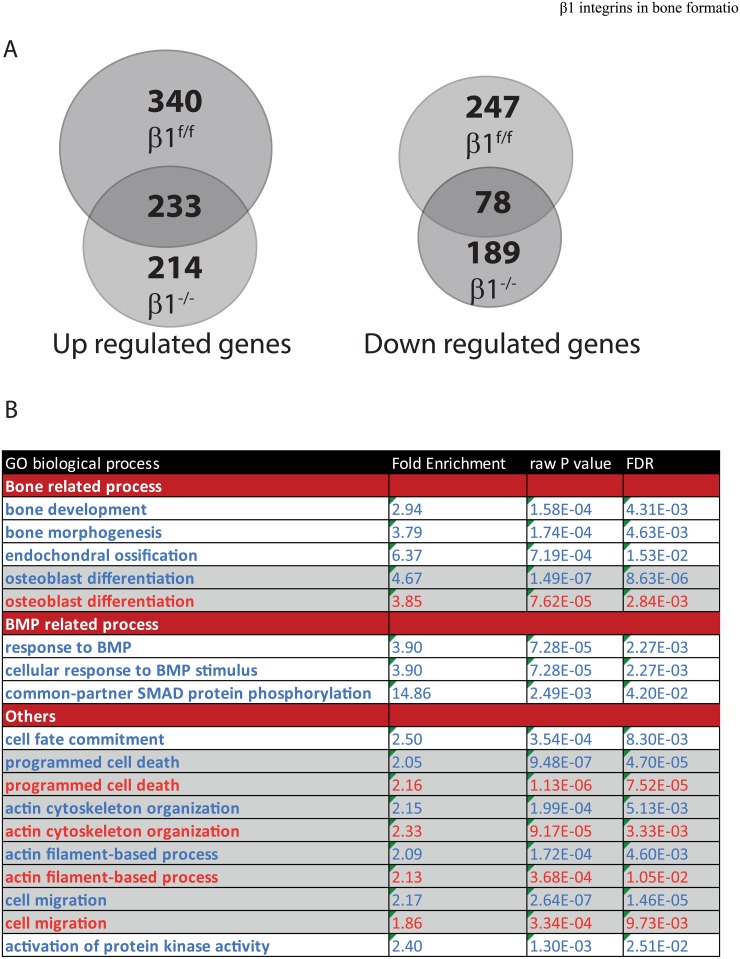
Transcriptomic analyses of BMP2 response. **A.** Diagrams representing the number of up and down regulated genes in control and β1 deficient cells in response to BMP2 stimulation, respectively. **B.** Amigo2 online software analysis of GO biological process modified upon BMP2 treatment in control (blue) and β1 deficient cells (red). Biological processes were selected based upon biological interest (bone related signature). FDR = False Discovery Rate.

Next, we performed an unbiased transcriptomic analysis from wild-type and β1 deficient cells upon BMP2 stimulation to disclose a broader view of how the loss of β1 integrins impacts the BMP2 response. In good agreement with the reported phosphorylations above and Smads nuclear localization, we noticed that the BMP2 response was not completely obliterated (Fig 6, S1 and S2 Tables). Indeed, we observed in control cells that out of the 897 genes that were regulated by BMP2, 572 were upregulated and 325 downregulated (S1 Table). In β1 deficient cells 714 genes were regulated by BMP2 with 447 genes upregulated and 267 genes downregulated (S2 Table). In the BMP2 induced gene response, there was a significant overlap with 233 common genes upregulated and 78 genes downregulated in both WT and β1 deficient genotypes (Fig 6A). While most genes that were modulated by BMP2 in both genotypes did not display any significant differences, few of them exhibited more than a two-fold in between control and β1 deficient cells difference (S3 Table). It is noteworthy, that some of these genes were described to be involved in osteoblast function or differentiation such as FGFR2, FN1 [11, 27]. Additionally, the analysis of modified biological processes, upon BMP2 treatment in both control and mutant cells using the online Amigo2 algorithm, further supported the idea that BMP2 induced osteoblastic signature was specifically affected by β1 integrin deficiency (Fig 6B). Indeed, gene signature related to bone development that was enriched in control cells upon BMP2 stimulation was no longer found in β1 deficient cells, and the overall signature corresponding to the osteoblastic differentiation was significantly reduced (enriched 4.67 fold in control versus 3.85 fold in β1 deficient cells).

Although the response to BMP2 appeared to be somehow preserved in β1 deficient cells, some important genes involved during osteoblastic differentiation were not induced or dramatically reduced such as osterix (SP7), type I and XII collagens (two collagens upregulated during osteogenesis) and Itga11 [28, 29]. In sharp contrast, Zfp521, a negative regulator of Runx2 was upregulated in β1 deficient cells [30]. Thus, this analysis further confirmed that β1 integrins play a pivotal role in the control of gene expression required for supporting a full osteoblastic differentiation, but likely not directly by regulating BMP2 response.

## Discussion

Herein, we provided evidence and confirmed that β1 integrins are required at the early stages of osteoblast differentiation *in vivo* and consequently for proper bone formation. We further demonstrated that β1 integrins influence the expression of BMP2 target genes by acting not at the BMP receptor level as suggested by earlier studies [9, 13, 23, 31] but rather downstream the BMP2 signaling pathway and SMADs activation/nuclear translocation, probably at the transcriptional level. These data underline the complexity of the regulation of osteoblast differentiation and exemplify the necessity for a cell to be at the right place at the right time in order to differentiate properly and become functional.

Integrins involvement in growth factor signaling has long been recognized [13, 23]. Surprisingly, *in vivo* and *ex vivo* results showed an apparent contradiction. Indeed, *in vivo* BMP2 signaling is affected at the level of SMAD1/5 phosphorylation whereas *ex vivo*, SMAD1/5 phosphorylation and nuclear translocation were not affected. Despite this, the expression of some of the BMP2 target genes were still blunted in the absence of β1 integrins. We can explain this apparent discrepancy by the fact that BMP2 expression is regulated by the BMP/SMAD pathway itself [32]. Thus, a defective response to BMP2 (at the transcriptional level) due to β1 integrin loss would lead to a reduced secretion of BMP2 in the microenvironment *in vivo*, consequently reducing BMPR and SMAD1/5 activation in osteoblasts. Hence, β1 integrins by regulating BMP-2 and -4 expression and secretion might indirectly regulate SMAD1/5 phosphorylation level *in vivo*. Supplementation of exogenous BMP2 *ex vivo* and consequently the independence of osteoblasts to their own BMP2 production may explain why p-SMAD1/5 defect was not observed under *ex vivo* experimental conditions. Alternatively, the nature of the extracellular matrix on which the osteoblasts adhere onto may also conciliate *in vivo* and *ex vivo* data. Indeed, osteoblasts express β1 integrin subunits that can associate with α1 and α5 and form functional receptor dimers for type I collagen and fibronectin (FN), respectively. On the other hand, they also express αv integrin subunit that associates to β3 or β5 to form FN or Bone Sialoprotein (BSP) receptors. Therefore, one can speculate that *in vivo* the ECM located close to differentiating osteoblasts does not allow the engagement of αv containing integrins by osteoblasts. In this case, the absence of integrin activation upon β1 integrin deletion leads to an impairment of BMP2 signaling at the receptor or SMAD1/5 level. In contrast *ex vivo* αv integrins may compensate the β1 integrin loss and rescues SMAD1/5 phosphorylation downstream BMPR activation. Indeed, our results suggest that integrins in general are necessary for proper BMPR activation and SMAD1/5 phosphorylation but that β1 integrins in particular are required for SMAD1/5-dependent transcriptional regulation of BMP2 target genes. Further experiments would be needed to address the role of αv vs β1 integrins and respective complementary matrices at different levels of BMP signaling.

It was nicely shown that osteoblast differentiation is finely regulated in vivo. The less differentiated cells being in a softer environment while mature osteoblasts being located on the rigid bony surface [9]. β1 integrins being one of the major osteoblast cell surface receptors involved in the matrix sensing, we can speculate that β1 removal might interfere with the osteoblast capacity to sense their extracellular environment. Indeed, our *in vivo* data highlight a critical role for β1 integrins in osteoblast function, while *in vitro* data supports that β1 integrins are required for a selective transcriptional regulation of BMP2 target genes. This appears to be downstream of Smad1/5 phosphorylation and nuclear translocation. How exactly this network is regulated by β1 integrins remains speculative, but we anticipate that β1 integrins regulate the formation of molecular complexes promoting an osteogenic specific transcription program. Supporting the hypothesis of a missing co-factor to cooperate with a BMP2 dependent gene expression, is the observation that β1 deficient cells are still responsive to a BMP2 stimulation. Since we recently reported that β1 is a key regulator of YAP nuclear localization, [33] we can speculate that YAP/TAZ signaling might be involved. Indeed, is was recently shown that YAP/TAZ co signal with Smads to commit cells into the bone lineage in a fish model [34]. Therefore, one can hypothesize that β1 integrins might control Smad dependent transcription through the regulation of the Smad co-transcription factor YAP or TAZ. Whatever the cofactor involved, it is noteworthy that under BMP2 stimulation the expression of the key osteogenic transcription factor osterix (a Runx2 target gene) was compromised in β1 deficient cells. As RUNX2 and osterix cooperate with Smad1/5 to activate the expression of BMP2 target genes, one can imagine that the defect observed in β1 deleted cells might be due to a RUNX2 or osterix transcriptional defect. This would explain the selective defect in the BMP2 response in β1 deficient cells. Clearly, further investigations will be needed to precisely decipher how β1 integrins influence BMP2-dependent transcription of osteogenic genes and whether YAP nuclear translocation might be involved in the establishment of this network, but our data clearly support that β1 integrin signaling is involved in the fine coordination of different inputs that led to the regulation of a specific gene expression pattern.

## Experimental procedures

### Mouse genetics

Mouse strain with floxed alleles of β1 integrin subunit (*Itgb1*^*tm1Ref*^) was kindly provided by Pr R. Fässler (Max Planck Institute, Martinsried, Germany). The *Osx1-GFP*:*Cre* deletor mouse was from Dr A. McMahon. Mice were generated in a mixed background (Sv129/C57B6). Mice were kept at Grenoble University Animal house facility under regular conditions of husbandry accordingly to the European rules and the project have been approved by the University Ethical committee (National ethical committee number 11, project number: APAfiS 10218). Mice were euthanized using CO_2_ asphyxia methods accordingly to the French legislation on animal research.

### Antibodies plasmids and reagents

Antibodies: Anti-phospho-ERK (#4370), -phosphop38 (#4831), phosphor-AKT (#4058), and—phospho-Smad1/5 (#9511) were from Cell Signaling (Ozyme, St Quentin en Yvelines France). Anti-β1 integrin (MB1.2) was from BD Biosciences (Le Pont de Claix, France). For IHC, β1 integrin clone 4B7R antibody (Abcam, Paris, France) was used. The anti-phosphotyrosine (PY) monoclonal antibody 4G10 used as hybridoma supernatant was produced in our laboratory. Anti-Actin (clone AC-40), anti -Type I Collagen and doxycycline were from Sigma-Aldrich (L’Isle d’Abeau, France). BMP2 was from Shenandoah Biotechnology Inc. and was used at a 200μg/mL concentration. pEl-BRE4xLuc plasmid was a gift from J. Massagué (NY, USA).

### Cell cultures and cell lines

Osteoblast cell lines were generated from newborn calvaria as previously described [11]. Cells were immortalized with a retrovirus expressing the large SV40 T antigen, and were maintained at 37°C with 5% CO2 in DMEM 10% FCS supplemented with penicillin and streptomycin. Immortalized β1^f/f^ cells were infected with an adenoviral supernatant encoding the Cre recombinase for 1h in PBS supplemented with 2% FCS and 1mM MgCl_2_. Osteoblast were cloned and β1 integrin deletion was checked by immunofluorescence.

### Histomorphometry and bone density measurements

Tibiae from 2-month old animals were fixed and embedded in methylmethacrylate. Sections were deplasticized and stained for Masson-Goldner with hematoxylin (Gill II), acid fuchsin/ponceau xylidine, and phosphomolybdic acid/orange G to stain the cells and osteoid, and light green to stain the mineralized matrix [35]. Primary cancellous bone was defined as the 120 μm band below the growth plate. Cancellous bone was defined as the remaining trabecular area that extends down 2 mm. The absolute osteoblast number in the cancellous bone was evaluated and reported. pQCT of the distal femur was performed with XCT Research SA (StraTec Medizintechnik). Trabecular BMD was measured at 9% of the bone length below the growth plate using peel-mode 20 [36].

### Skeletal staining

Mice skeletons were fixed in 70% ethanol during 2 days, transferred to acetone 2 more days, then stained 2 days in staining solution (Alizarin Red 0.05g/l, Alcian Blue 0.15g/l, acetic acid 5% v/v). Finally, remaining soft tissues were digested in 1% KOH.

### Immunofluorescence and TRAP staining

Cells were fixed with 4% paraformaldehyde-PBS for 15min. Following permeabilization and blocking with goat serum, cells were incubated with primary antibodies during 1 hour. Secondary antibodies used were conjugated with Alexa 488 and Alexa 555 from Jackson Immunoresearch (Interchim, Montluçon, France). Samples were mounted using Mowiol 4–88 reagent (Sigma Aldrich) supplemented with DAPI (Life technologies, St Aubin, France) and were analyzed using an upright Axioimager M2 microscope (Carl Zeiss SAS, Le Pecq, France.).

For paraffin-embedded tissues, sections were prepared and immunostained following deparaffinization and hydration. TRAP stainings were performed using the TRAP kit from Sigma Aldrich and according to the manufacturer’s instructions.

### Bone and cell lysates and Western-blotting

Protein lysates from bones were obtained from mice long bones carefully cleaned of any muscle and bone marrow. Bone from 3 mice were pooled and frozen at -80°C then crushed by shaking in presence of steel ball (Retsch MM400; 3 cycles of 1 minute at 30 movements/s) and lysed in RIPA lysis buffer containing proteases and phosphatases inhibitors. Cells were lysed using RIPA lysis buffer containing proteases and phosphatases inhibitors. Lysates from both bone and cell culture were centrifuged at 15000rpm for 30 min at 4°C, and supernatants were used for immunoblotting using standard protocol. Experiments were repeated two times.

### Osteoblast differentiation

*In vitro* differentiation of isolated osteoblasts was performed essentially as previously described [12]. Briefly, 60,000 cells per well were plated in a 24-well tray. After 3 days of culture, when cells were confluent, the medium was switched to differentiation medium (α-MEM, 10% FCS, 50 μg/ml ascorbic acid, 10 mM β-glycerophosphate) and changed every other day. The differentiation process was visualized by Alizarin Red S staining for calcium deposition as described previously [15].

### RNA extraction, Reverse transcription, Real-Time PCR and transcriptomic

RNA samples were prepared and analyzed as previously described (Brunner et al., 2011). Mouse primers were the following: RUNX2 forward, 5’- CCGCACGACAACCGCACCAT-3’; and reverse 5’- CGCTCCGGCCCACAAATCTC-3’; ALP forward, 5’- GCCCTCTCCAAGACATATA-3’ and reverse 5’- CCATGATCACGTCGATATCC-3’; OCN forward, 5’- AAGCAGGAGGGCAATAAGGT-3’ and reverse 5’- AGCTGCTGTGACATCCATAC-3’; Osx forward, 5’- CCTAGCAGACACCATGAG-3’ and reverse 5’- TCTGATAGCTCGTCACAAG-3’.

Total RNA was isolated from β1^f/f^ and β1^-/-^ immortalized osteoblast and purified using TRIzol reagent (Thermo Fisher Scientific, Waltham, USA) and RNeasy Kit (Qiagen, Courtaboeuf, France) following the manufacturer’s instructions. Total RNA quantification was performed using the Nanodrop ND- 1000 spectrophotometer (Thermo Fisher Scientific, Waltham, USA). RNA was reverse-transcribed with the iScript Reverse Transcription Supermix (Biorad, Hercules, USA). Real-time qPCR analysis was performed using iTaq Universal SybrGreen Supermix (Biorad, Hercules, USA) on Biorad CFX96.

The integrity of the extracted RNAs was assessed with the Bioanalyzer 2100 and the RNA6000 Nano kit (Agilent Technologies Incorporation, Santa Clara, USA). A RNA integrity number (RIN) greater or equal to 7.00 was achieved for all samples. No sign of DNA contamination was detected in any of the samples analyzed. The starting amount of total RNA used for the reactions was 400 nanograms per sample, for all samples. The Illumina Total Prep RNA Amplification Kit (Applied Biosystems / Ambion, Austin, USA) was used to generate biotinylated, amplified cRNA according to the manufacturer recommendations. Hybridization, staining and detection of cRNAs on Illumina Mouse WG-6 v2 Expression BeadChips were performed according to the manufacturer’s protocol. The MouseWG-6 v2.0 BeadChip profiles more than 45,200 transcripts derived from the National Center for Biotechnology Information Reference Sequence (NCBI RefSeq) database (Build 36, Release 22), the Mouse Exonic Evidence Based Oligonucleotide (MEEBO) set as well as from exemplar protein-coding sequences described in the RIKEN FANTOM2 database. The Illumina I-Scan system was used to scan all Expression BeadChips, according to Illumina recommendations.

Using the Gene Expression Module 1.9.0 of GenomeStudio V2011.1 software (Illumina—USA), the Quantile normalization method was applied to the primary probe data. Processed probe data were then filtered according to the following criteria: minimal signal intensity fold change of 1.50 across all samples, minimal probe signal intensity absolute change of 150 across all samples. Filtered data were then log2-transformed, and the expression values compared between the β1^-/-^cells and wild-type β1^f/f^ samples using Omics Explorer 3.2(42) (Qlucore, Sweden). Genes were considered differentially expressed when their expression level satisfied two criteria: the adjusted p-value (q-value) was < 0.01(which corresponded to a |R|> 0.96 ii) the absolute fold change between the mean expression value in the samples from mutant cells compared to that in controls was > 1.5.

### Bioinformatic analysis

Gene ontology analysis was performed using Amigo2 online software (http://amigo2.berkeleybop.org/amigo/landing) on selected genes. BMP2 regulated genes in control (897) and β1 integrin deficient cells (714) were submitted to the Amigo2 Term Enrichment Process. Biological process answers were analyzed according to web site specifications. Relevant processes were collected based on implication in BMP2 driven processes and a cut-off value of 1.6 fold enrichment was chosen as filter. Raw data analysis is provided as S4 Table.

## Supporting information

S1 Table Gene list up- and downregulated upon BMP2 in β1^f/f^ osteoblasts. (XLSX)

S2 Table Gene list up- and downregulated upon BMP2 in β1^-/-^ osteoblasts. (XLSX)

S3 Table BMP2 induced shared gene list in β1^f/f^ with β1^-/-^ osteoblasts. (XLSX)

S4 Table Raw data analysis of Amigo2 on β1^f/f^ with β1^-/-^ osteoblasts upon BMP2. (XLSX)

## Abbreviations

BSP: bone sialoprotein
ECM: extracellular matrix
FN: fibronectin
ICAP-1α: Integrin Cytoplasmic domain Associated Protein-1α
OCN: osteocalcin
Ost: Osterix
Osx: Col1: type I collagen
